# Mice Deficient in Nucleoporin Nup210 Develop Peripheral T Cell Alterations

**DOI:** 10.3389/fimmu.2018.02234

**Published:** 2018-09-28

**Authors:** Annemarie van Nieuwenhuijze, Oliver Burton, Pierre Lemaitre, Alice E. Denton, Ana Cascalho, Rose E. Goodchild, Bert Malengier-Devlies, Bénédicte Cauwe, Michelle A. Linterman, Stephanie Humblet-Baron, Adrian Liston

**Affiliations:** ^1^VIB Centre for Brain & Disease Research, VIB, Leuven, Belgium; ^2^Department of Microbiology and Immunology University of Leuven, Leuven, Belgium; ^3^Babraham Institute, Babraham Research Campus, Cambridge, United Kingdom; ^4^Department of Neurosciences, University of Leuven, Leuven, Belgium

**Keywords:** Nup210, CD4-positive T-lymphocytes, CD8-positive T-lymphocytes, thymus gland, spleen, nucleopore

## Abstract

The nucleopore is an essential structure of the eukaryotic cell, regulating passage between the nucleus and cytoplasm. While individual functions of core nucleopore proteins have been identified, the role of other components, such as Nup210, are poorly defined. Here, through the use of an unbiased ENU mutagenesis screen for mutations effecting the peripheral T cell compartment, we identified a Nup210 mutation in a mouse strain with altered CD4/CD8 T cell ratios. Through the generation of Nup210 knockout mice we identified Nup210 as having a T cell-intrinsic function in the peripheral homeostasis of T cells. Remarkably, despite the deep evolutionary conservation of this key nucleopore complex member, no other major phenotypes developed, with viable and healthy knockout mice. These results identify Nup210 as an important nucleopore complex component for peripheral T cells, and raise further questions of why this nucleopore component shows deep evolutionary conservation despite seemingly redundant functions in most cell types.

## Introduction

An understanding of the genetic requirements for T cell development has been built upon the analysis of murine and human T cell immunodeficiencies. These studies have identified genes that have roles in differentiation, function, maintenance, or homeostasis of T cells, with mutation leading to loss of the T cell population (1). While most mutations leading to T cell-deficiency have clear lineage-specific functions, there is a fascinating class of mutations in genes that are widely expressed and have basic cell-biological functions, such as gene regulation [DNMT3β, SP110 (2, 3)], chromatin remodeling [SMARCAL1 (4)], and metabolism [adenosine deaminase, nucleoside phosphorylase (5, 6)]. While complete loss of many of these genes would be anticipated to result in embryonic lethality, based on critical functions in cell biology, identified mutations tend to have T cell-specific defects. This observation is thought to be a result of selection bias, where only those point mutations mild enough to retain sufficient function for most cells result in viable offspring. It is not clear why T cells are sensitive to mild mutations that other cells can tolerate, however this may be related to the rapid rate of proliferation of the early stages of T cell differentiation (7, 8). Regardless, in multiple cases T cells have functioned as the “canary in the coal mine,” acting as a phenotyping read-out for mild mutations in critical cell biology genes.

Nup210 (or gp210) was the first nucleopore-associated protein to be discovered, and was initially thought to promote the fusion of inner and outer nuclear membranes during nucleopore assembly (9, 10). However, Nup210 is not ubiquitously expressed in all tissues, and the analysis of nucleopore complex (NPC) composition during mouse embryogenesis and in naturally Nup210-deficient cell lines showed that Nup210 is dispensable for the assembly or stability of the nucleopore complex (11–14). While this result may discard Nup210 as an essential component of the NPC, its symmetrical localization as a membrane ring around the nuclear pore, which is also observed for the yeast homolog Pom152 (15), and high conservation across eukaryotes, was suggestive of an important function in cell biology. More recently, it was shown that shRNA knockdown of Nup210 in myoblasts and embryonic stem cells induced apoptosis and completely abrogated their differentiation into myotubes and neuroprogenitor cells (16). Further studies have suggested that Nup210 is acting as a scaffolding protein for transcriptional complexes such as Mef2C, and that the tissue-specific expression is most likely a driver for the specialization of NPCs in different cell types, thereby playing a role in the regulation of cell fate (17). The regulatory role of the NPC in the import of transcription factors in T cells in the context of inflammation and immunity has been shown in a number of studies (18–21), and this cellular function has also been studied in detail in myocyte culture (17), however the role of NUP210 at the organism level has not previously been studied.

The dual identity of T cells as both critical coordinators of the immune system and also highly sensitive indicators of disturbed cellular processes makes them attractive targets for unbiased genome-wide genetic screens. Here we used an ENU mutagenesis screen for altered peripheral T cell phenotypes to identify an I476T point mutation in Nup210 which skews the CD4:CD8 compartment ratio. Generation of Nup210 knockout mice validated a T cell-intrinsic function for Nup210. The surprising viability of Nup210 knockout mice leads to the perplexing question of the driver of deep evolutionary conservation of a seemingly largely redundant nuclear pore factor.

## Results

### Mutation in conserved nucleoporin Nup210 alters composition of the T cell compartment

**Figure 1:**
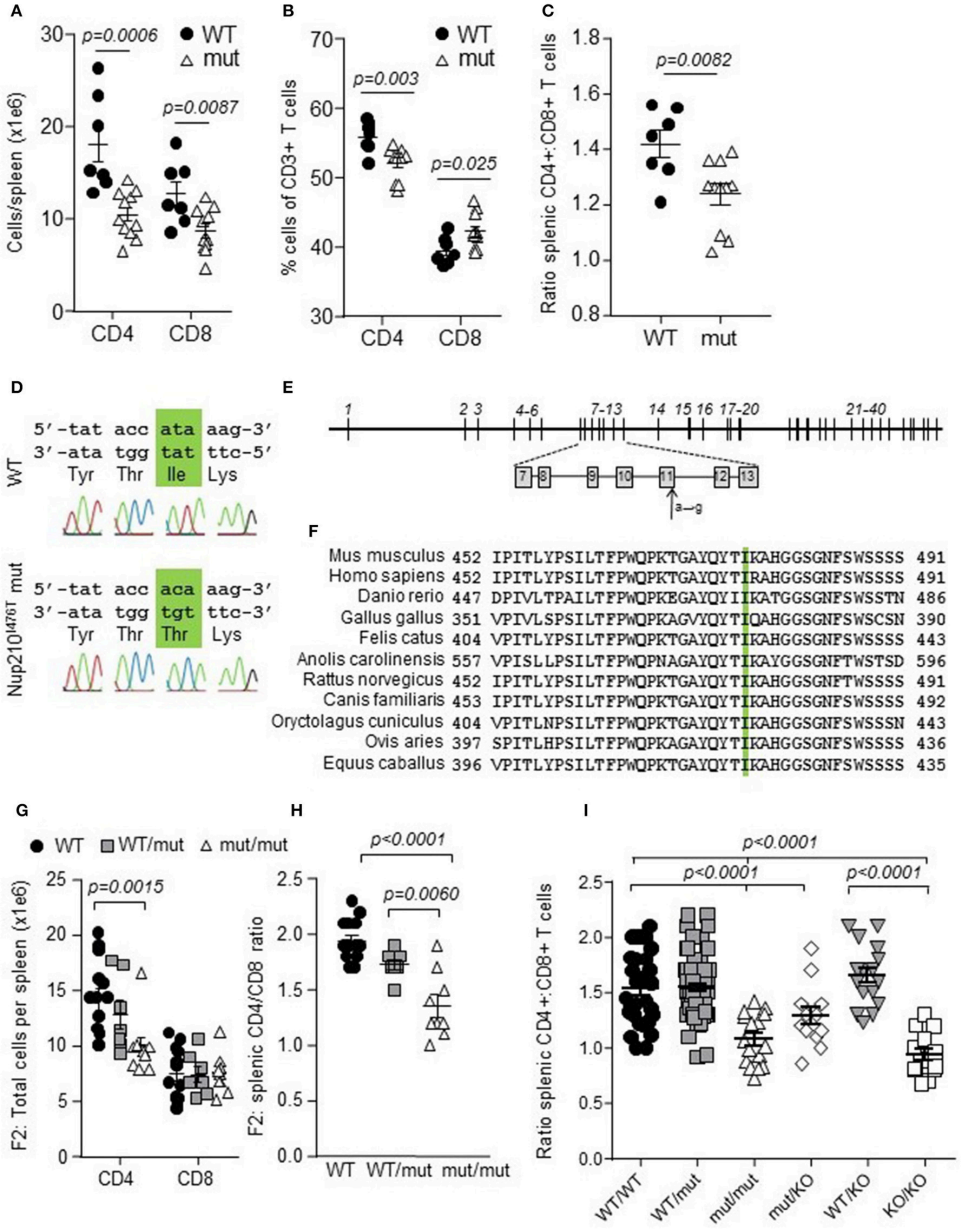
Altered ratio of peripheral CD4:CD8 T cells in Nup210 mutant mice. ENU mutagenesis generated a *Nup210*^*I*476*T*^ mouse strain, identified by peripheral blood screening for T cell composition. **(A)** Absolute numbers of cells in spleens from 5 to 6 week old wildtype (WT) and Nup210^I476T^ mutant (mut) mice. **(B)** Percent of splenic CD4 and CD8 T cells assessed by flow cytometry. **(C)** Ratio of splenic CD4 and CD8 T cells (*n* = 7, 11). **(D)** Sanger sequencing of *Nup210* in WT and Nup210^I476T^ mutant mice confirmed an A to G mutation, resulting in an isoleucine to threonine change at amino acid 476. **(E)** Schematic overview of the 40 exons of the *Nup210* gene, including the location of the I476T mutation in exon 11 (arrow). **(F)** Conservation of the mutation site between the mouse, human, zebrafish, chicken, cat, lizard, rat, dog, rabbit, sheep, and horse homologous sequences. **(G,H)** Confirmation of germline transmission of the *Nup210*^*I*476*T*^ mutation in F2 offspring of the *Nup210*^*I*476*T*^ mutant founder mouse and resulting splenic phenotype in absolute cell numbers per spleen **(G)** and CD4:CD8 ratio **(H)** (*n* = 14, 7, 9). **(I)** Replication of the *Nup210*^*I*476*T*^ mutant phenotype (spleen) in a complementation cross (*n* = 33, 71, 16, 16, 20, 17). Mean ± SEM, with individual biological replicates.

As part of an unbiased screen for genetic control of the peripheral T cell compartment, N-ethyl-N-nitrosourea (ENU)*-*exposed C57BL/6 mice were bred to *Foxp3*^*GFP*^ females to generate a standard F2 intercross pedigree (22). The inclusion of *Foxp3*^*GFP*^ allowed changes to the regulatory T cell (Treg) compartment to be included in the screening protocol. The resulting ENU mutants were screened for altered ratios of CD4 and CD8 T cells in the peripheral blood and spleen. Within one pedigree, individuals were identified with a decreased CD4:CD8 ratio in the peripheral blood. Intercrossing of affected individuals resulted in a mutant strain which consistently demonstrated decreased CD4+ cells and a decreased CD4:CD8 ratio in the spleen at 5–6 weeks of age (Figures 1A–C). All-exon sequencing of affected individuals identified an A → G nucleotide substitution at nucleotide 1469 of the Nup210 gene, which was confirmed by Sanger sequencing (Figure 1D). This mutation in exon 11 (Figure 1E) resulted in a predicted isoleucine to threonine change at amino acid 476 (Nup210^I476T^). The mutation was located in an invariant amino acid in a region of Nup210 highly conserved throughout vertebrates (Figure 1F).

Nup210 is thought to be a key component of the nuclear pore complex (NPC), forming a membrane ring around the NPC (15). Due to the lack of known biology linking Nup210 to T cell-specific processes, we sought to validate the mutation through an F2 phenotyping cross and a complementation cross. First, *Nup210*^*I*476*T*^ mice were crossed with wildtype mice to produce an F1 generation and then intercrossed to produce an F2 generation. Genotyping for the *Nup210*^*I*476*T*^ allele allowed identification of wildtype, heterozygous and homozygous mice, while other, unlinked, ENU-induced alleles would be randomly segregated across the pedigree. Phenotyping of the F2 cross found a replication of the original finding, a reduction in CD4+ T cells and the CD4:CD8 ratio in *Nup210*^*I*476*T*^ homozygous mice (Figures 1G,H Supplementary Figure 1). Second, for a complementation cross, mice bearing a Nup210KO allele were generated using EUCOMM ES cells, with an insertion of a LacZ/Neo cassette between exons 3 and 5 (Supplementary Figure 3). Mice bearing one copy of the *Nup210*^*I*476*T*^ allele and one copy of the *Nup210*^*KO*^ allele phenocopied the *Nup210*^*I*476*T*^ homozygous mice (Figure 1I). Together, these data validated Nup210 as a novel genetic control mechanism controlling CD4:CD8 ratio.

### Nup210 knockout mice are viable and have intact thymic development

**Figure 2:**
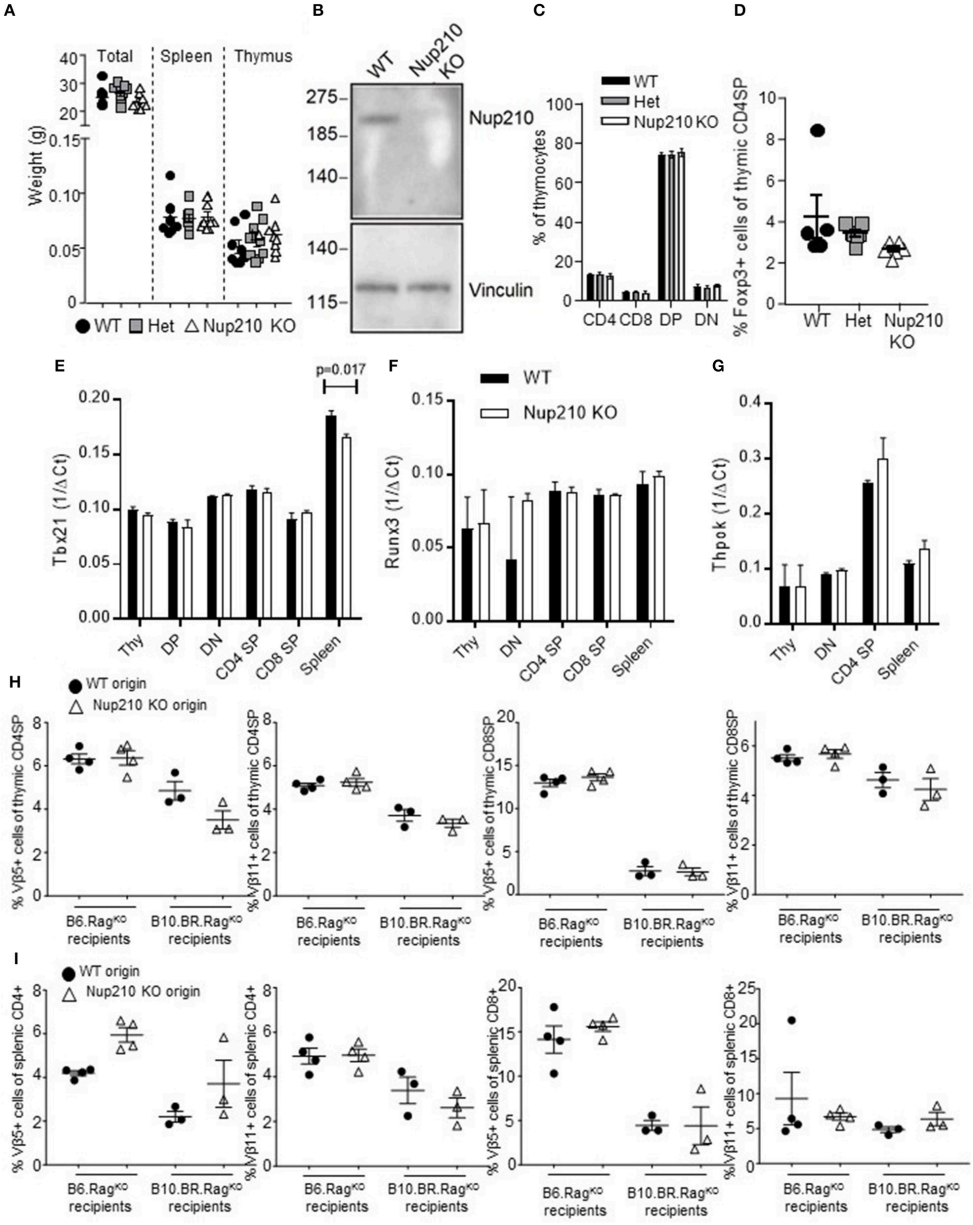
Nup210 knockout mice are viable and have intact thymic biology. **(A)** Six to 11 week old wildtype, *Nup210*^*het*^, *Nup210*^*KO*^ mice were analyzed for body weight, spleen weight and thymus weight (*n* = 8, 9, 9). **(B)** Western blot for Nup210 in the lysates of wildtype and *Nup210*^*KO*^ thymocytes. Vinculin was used to control for protein input (representative of 2 independent experiments). **(C,D)** Wildtype, *Nup210*^*het*^, *Nup210*^*KO*^ mice (*n* = 5, 7, 6) were assessed by flow cytometry for **(C)** double negative (DN), double positive (DP) and CD4 and CD8 single positive subsets in the thymus; **(D)** Foxp3+ regulatory T cells in the thymic CD4 compartment. **(E–G)** Wildtype and *Nup210*^*KO*^ mice were used as donors for whole thymocytes, DN, DP, CD4 SP, CD8 SP, and whole splenocytes. Normalized expression of *Tbx21, Runx3*, and *Thpok* in each population (only plotted when mRNA could be detected), through qPCR (*n* = 2 mice per genotype; representative result from *n* = 3 experiments). **(H)** Bone-marrow from CD45.1 wildtype and CD45.2 *Nup210*^*KO*^ mice was mixed at a 1:1 ratio and used to reconstitute irradiated B6.Rag^KO^ and B10.BR.Rag^KO^ recipients. Mice were assessed in both the thymus and **(I)** spleen for the proportion of Vβ5- and Vβ11-expressing CD4 and CD8 T cells. Mean ± SEM, with individual biological replicates.

While no known functions of Nup210 are linked specifically to T cell biology, the rapid rate of proliferation of early stage thymocytes (double negative T cells) is known to sensitize this lineage to minor genetic insults in general cell biology components, such as the chromatin condensing unit kleisin beta (7). Based on the essential function of Nup210 in the nuclear pore complex, and the lethal phenotype that results from knock-down of Nup210 in cell lines (23), it was assumed that the *Nup210*^*I*476*T*^ allele was a mild hypomorph, with sufficient function maintained to prevent cell death in lineages beyond early T cell stages. The generation of mice bearing the *Nup210*^*KO*^ allele allowed direct testing of this hypothesis, by intercrossing *Nup210*^*het*^ mice to create *Nup210*^*KO*^ mice. Surprisingly, intercross of *Nup210*^*het*^ mice produced wildtype:*Nup210*^*het*^:*Nup210*^*KO*^ mice at the Mendelian 1:2:1 ratio. *Nup210*^*KO*^ mice were viable, demonstrated no visual abnormalities or altered total bodyweight or the weight of key immunological organs (Figure 2A). Histological screening of the organs was unremarkable (data not shown). Complete knock-out of Nup210 in *Nup210*^*KO*^ mice was confirmed by Western blot (Figure 2B), leaving the perplexing finding that an evolutionarily conserved component of the nuclear pore complex is largely redundant for life. We therefore generated mouse embryonic fibroblast (MEF) lines from wildtype and *Nup210*^*KO*^ littermates to allow for nucleopore imaging. Fibroblasts were stained with mAb414, which detects a number of FXFG-repeat-containing nucleoporins, such as Nup62, Nup153, Nup214, and Nup358/RanBP2. Confocal imaging revealed that the frequency and distribution of nuclear pores between the two genotypes was similar (Supplementary Figure 2). Nup210 does not, therefore, have non-redundant functions in nucleopore formation.

Thymocytes are highly susceptible to cellular stress, owing to the rapid proliferation at the double negative (DN) stage of development in the thymus, followed by the precarious double positive (DP) stage, at which “death by neglect” is the default outcome (1). We therefore tested whether the peripheral T cell phenotype observed in *Nup210*^*KO*^ mice is due to thymic defects. No difference was observed between wildtype and knockout mice in the DN or DP stages, or in single positive (SP) thymocytes which had diverged into either the CD4 or CD8 lineage (Figure 2C), or Foxp3+ regulatory sublineage (Figure 2D). Key transcription factors known to impact the CD4-CD8 lineage decision in the thymus, Tbx21, Runx3, and Thpok, remained unchanged throughout thymic differentiation (Figures 2E–G). In order to test the efficiency of negative selection, we utilized the endogenous MMTV self-superantigen, which recognizes both Vβ5 and Vβ11 TCR and I-E MHC molecules with a high affinity (24). Using a mixed bone-marrow chimeric system, of CD45.1 wildtype bone-marrow and CD45.2 *Nup210*^*KO*^ bone-marrow, we reconstituted B6.*Rag*^*KO*^ mice and B10.BR.*Rag*^*KO*^ mice. As B6 mice do not express I-E, MMTV can only drive TCR signaling weakly, allowing the Vβ5- and Vβ11-bearing T cells to pass through negative selection intact (Figures 2H,I). By contrast, in B10.BR mice, Vβ5- and Vβ11-bearing T cells receive a strong TCR signal from I-E, and undergo efficient negative selection (Figures 2H,I), a system which allows the detection of genetic modifiers of negative selection efficiency (25). In comparison to wildtype thymocytes, Nup210-deficient thymocytes demonstrated efficient negative selection (Figures 2H,I). *Nup210*^*KO*^ mice, in summary, demonstrate largely intact thymic development, through early differentiation, positive selection, CD4-CD8 lineage separation and negative selection. Together, these results demonstrate that the role of Nup210 in shaping the peripheral T cell compartment is a novel, peripheral-specific function, rather than a reflection of thymic sensitivity to basic cellular processes.

### Nup210-deficiency leads to an altered peripheral T cell compartment

**Figure 3:**
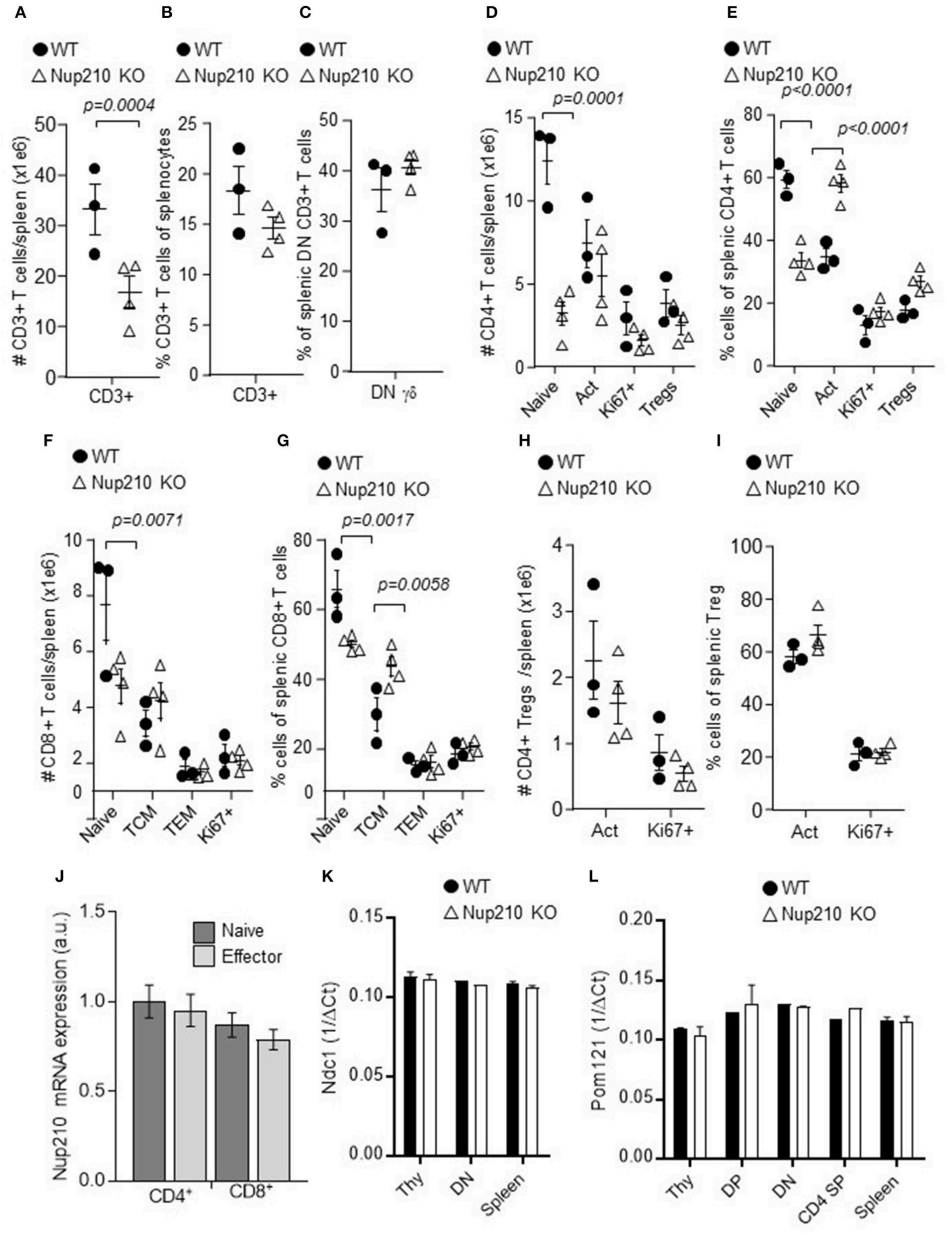
Altered peripheral T cell biology in Nup210 knockout mice are viable and have intact thymic biology. Wildtype and *Nup210*^*KO*^ mice (*n* = 3, 4) were assessed by flow cytometry for absolute and relative numbers of CD4 and CD8 T cell subset in the spleen. **(A)** Absolute number and **(B)** percentage of CD3+ T cells. Proportion of γδ T cells within the CD3+CD4-CD8- double negative (DN) population. **(D)** Absolute number and **(E)** percentage of CD4 T cell subsets, including naïve, activated (Act), Ki67^+^, and Foxp3^+^ (Tregs). **(F)** Absolute number and **(G)** percentage of CD8 T cell subsets, including naïve, central memory (TCM), effector memory (TEM) and Ki67^+^. **(H)** Absolute number and **(I)** percentage of Treg subsets, including activated (Act) and Ki67+. Mean ± SEM, with individual biological replicates. **(J)** Naïve and effector CD4 and CD8 T cells were sorted from wildtype mice and Nup210 mRNA was assessed by qPCR. **(K)** Wildtype and *Nup210*^*KO*^ mice were used as donors for whole thymocytes, DN, DP, CD4 SP, CD8 SP, and whole splenocytes. Normalized expression of *Ndc1* and **(L)** *Pom121* in each population (only plotted when mRNA could be detected), through qPCR (*n* = 2 mice per genotype; representative result from *n* = 3 experiments).

Intact thymic development in *Nup210*^*KO*^ mice indicates that the CD4-CD8 ratio skew initially observed in *Nup210*^*KO*^ mice results from peripheral abnormalities. Both the myeloid compartment (Supplementary Figure 4) and B cell compartment (Supplementary Figure 5) were largely unaltered. We therefore investigated the peripheral T cell compartment in greater detail (Supplementary Figure 6). *Nup210*^*KO*^ mice showed a near-50% reduction in total T cell numbers (Figure 3A), less notable in percentages (Figure 3B). γδ T cells were largely intact, with no alteration in the frequency (Figure 3C). Within the αβ T cell lineage, *Nup210*^*KO*^ mice demonstrated the greatest change in naïve CD4 T cells, which were reduced by 70% in absolute numbers in the spleen (Figure 3D). While other CD4 T cell subsets were intact in absolute numbers, the reduction in naïve T cells was reflected in a proportional increase in activated T cells (Figure 3E). CD8 T cells were affected in a similar way, with a 50% reduction in naïve CD8 T cell numbers (Figure 3F), and corresponding increases in proportion of central memory (TCM) cells (Figure 3G). Tregs (Figure 3H) and Treg subsets (Figure 3I) were largely intact. The relatively mild decrease in naïve CD8 T cells, compared to naïve CD4 T cells, is sufficient to explain the initial CD4-CD8 ratio skew observed (Figure 1I). The molecular basis for the differential CD4 T cell compared to CD8 T cell response was not due on differential expression of Nup210 (in wildtype mice) (Figure 3J), and compensatory upregulation of two other scaffolding proteins, Pom121 and Ndc1, was not increased in response to loss of Nup210 (Figures 3K,L). Together, these results demonstrate that the role of Nup210 in shaping the peripheral T cell compartment is a novel, peripheral-specific function, rather than a reflection of thymic sensitivity to basic cellular processes.

**Figure 4:**
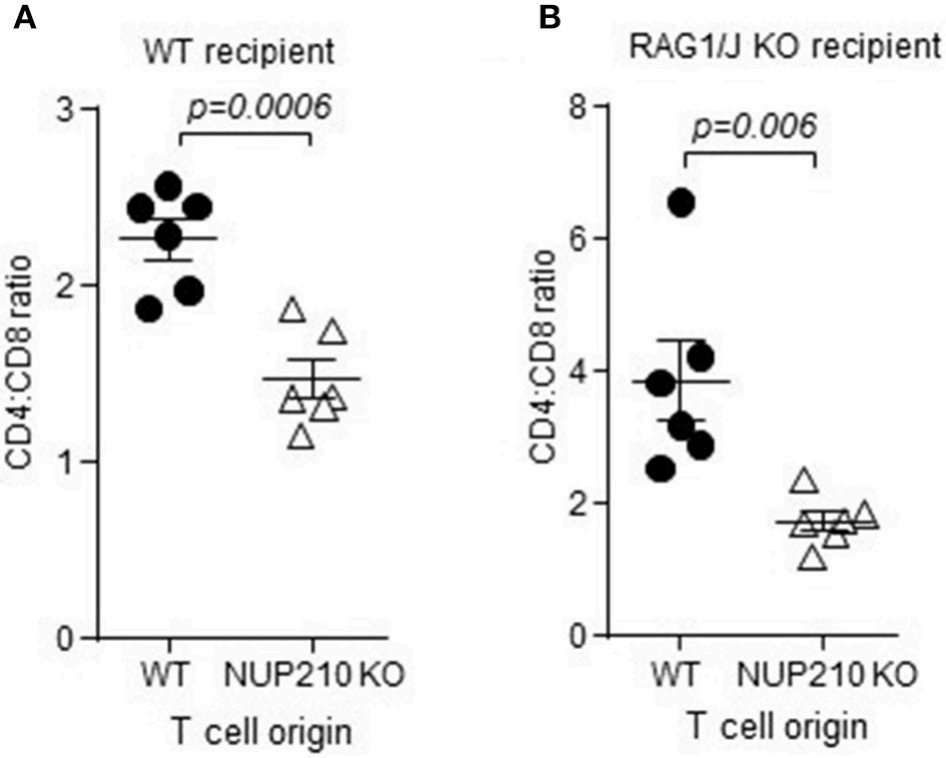
Altered ratio of peripheral CD4:CD8 T cells in *Nup210*^*KO*^ mice is due to functions intrinsic to T cells. **(A)** Wildtype CD45.1 mice were irradiated and reconstituted with 50% bone-marrow from wildtype CD45.1 mice and 50% bone marrow from *Nup210*^*KO*^ CD45.2 mice (*n* = 6). At 8 weeks post-reconstitution, the CD4:CD8 ratio in the spleen was assessed by flow cytometry in both the wildtype (CD45.1+) and *Nup210*^*KO*^ (CD45.2+) compartment. **(B)** *Rag1*^*KO*^ CD45.2 mice were irradiated and reconstituted with 50% bone-marrow from wildtype CD45.1 mice and 50% bone marrow from *Nup210*^*KO*^ CD45.2 mice (*n* = 6). At 8 weeks post-reconstitution, the CD4:CD8 ratio in the spleen was assessed by flow cytometry in both the wildtype (CD45.1+) and *Nup210*^*KO*^ (CD45.2+) compartment. Mean ± SEM, with individual data points.

Further investigation into the function of Nup210 in T cells was led by compartment analysis. Mixed bone-marrow chimeras were set up, where wildtype and *Nup210*^*KO*^ haematopoietic stem cells reconstitute an irradiated mouse. This approach allows the competitive comparison of wildtype and *Nup210*^*KO*^ T cells in a context where the host environment is directly shared, thus excluding any effects of non-haematopoietic origin. Furthermore, the mixed bone-marrow chimera approach allows intrinsic versus extrinsic differences to be identified, as extrinsic differences act in trans and are shared across bone-marrow origin, and only cis-acting intrinsic differences allow an origin-dependent phenotype. Using this system, the CD4:CD8 ratio disturbance still developed in *Nup210*^*KO*^ cells in this context (Figure 4A), demonstrating that the function of Nup210 in driving this phenotype is intrinsic to T cells. The same result was also demonstrated when reconstituting Rag-deficient mice, where contamination from residual host-derived T cells can be excluded (Figure 4B). These results indicate a T cell-intrinsic phenotype driven by Nup210-deficiency.

**Figure 5:**
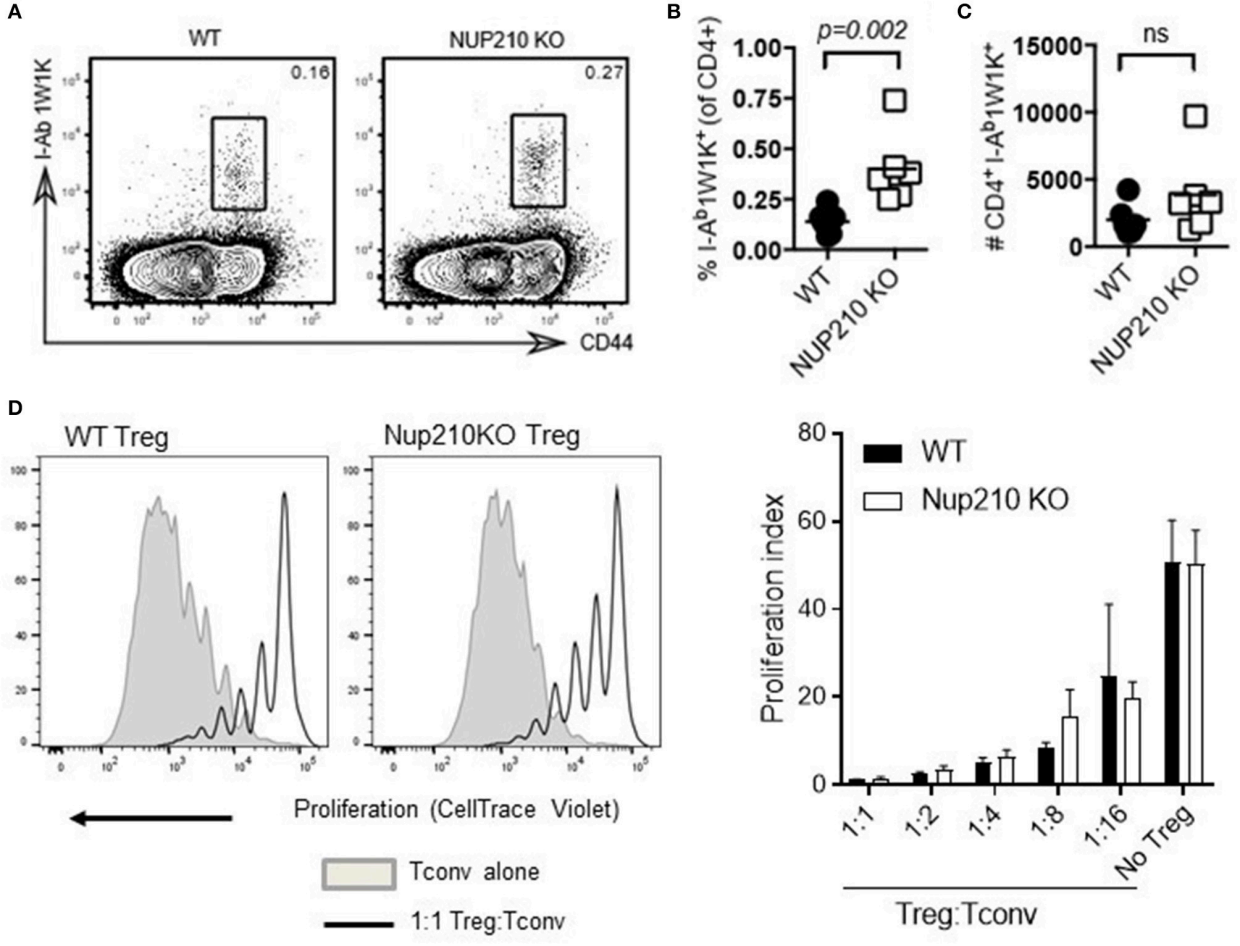
Nup210-deficient T cells exhibit intact immunogenic and tolerogenic properties. **(A)** WT and *Nup210*^*KO*^ mice were immunized subcutaneously with 1W1K peptide emulsified in IFA and the generation of I-A^b^ 1W1K-specific CD4^+^ T cells was determined 8 days later by tetramer staining. Representative flow cytometry profiles of I-A^b^ 1W1K tetramer and CD44 staining, gated on CD4^+^ T cells. These data were used to determine the proportion **(B)** and number **(C)** of I-A^b^1W1K-specific CD4^+^ T cells. Data are representative of one experiment with 6 mice per group; each data point represents an individual animal. Statistical significance was determined using a Mann-Whitney rank test; exact *p*-values are shown for significant differences. **(D)** CD4^+^CD25^+^ regulatory T cells were sorted from wildtype and Nup210KO mice and incubated at increasing ratios with wildtype naïve CD4 T cells (Tconv). Representative CellTrace Violet dilution histograms are shown (left), along with the proliferation index (right), a measure of the median number of divisions.

The demonstration that the function of Nup210 was T cell-intrinsic led to further fine phenotyping of *Nup210*^*KO*^ mice. Using the 1W1K immunization system, antigen-specific T cell responses were assessed (Figure 5A). While *Nup210*^*KO*^ mice showed an increased percentage of 1W1K-reactive T cells (Figure 5B), the absolute magnitude of the response was normal (Figure 5C), indicating that antigen-specific T cell responses were intact. Regulatory T cell responses were assessed through *in vitro* suppressive assays, where purified Tregs from *Nup210*^*KO*^ mice showed a normal level of suppressive function (Figure 5D). These results indicate that basic immunogenic and tolerogenic processes are intact in Nup210-deficient T cells.

**Figure 6:**
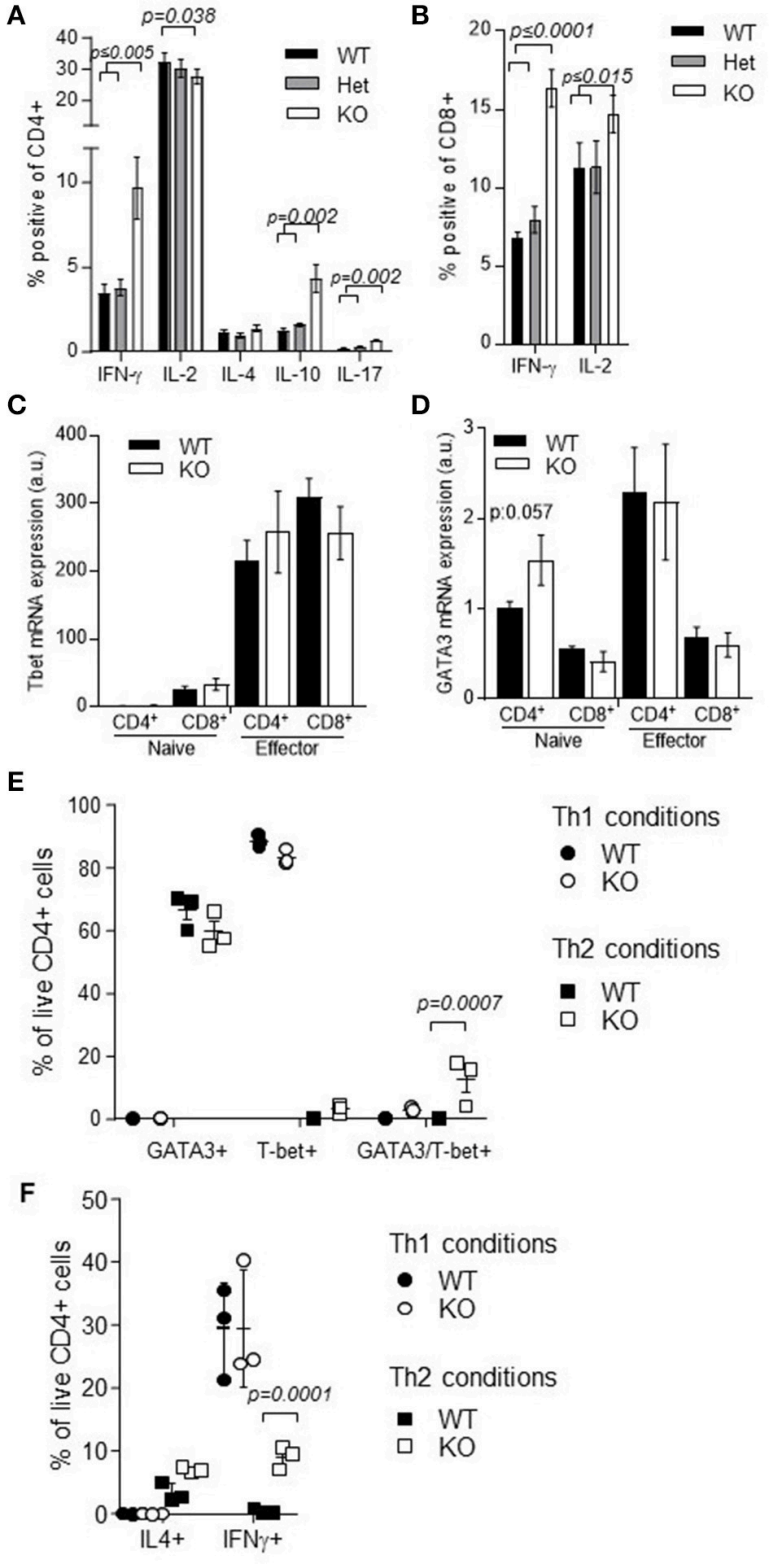
*Nup210*^*KO*^ mice exhibit an inflammatory T cell signature. Splenocytes from wildtype, *Nup210*^*het*^, *Nup210*^*KO*^ mice (*n* = 6, 7, 5) were assessed by flow cytometry. **(A)** Cytokine production by CD4+ cells or **(B)** CD8+ cells after PMA/ionomycin stimulation. **(C)** Naïve and effector CD4 and CD8 T cells were sorted from wildtype mice and Tbet and **(D)** GATA3 mRNA was assessed by qPCR. **(E)** Naïve CD4 T cells were purified from wildtype and Nup210KO mice and incubated *in vitro* under Th1- or Th2- polarizing conditions. The proportion of cells expressing GATA3, Tbet or both GATA3 and Tbet was assessed by flow cytometry on day 3, and **(F)** on day 6, the proportion of cells expressing IL-4 and IFNg was likewise assessed. Statistical significance was determined using a Mann–Whitney rank test; exact *p*-values are shown for significant differences.

In contrast to the basic immunogenic and tolerogenic processes, Th1 and Th2 responses were substantially altered in *Nup210*^*KO*^ mice. Compared to heterozygous littermates, *Nup210*^*KO*^ mice manifested increased numbers of IFNγ-producing CD4 and CD8 cells after stimulation, while Th2 and Th17 cells were unchanged (Figures 6A,B). These results at the cellular level were disparate from at the transcriptional level, where *ex vivo* T cells demonstrated normal *Tbet* (Figure 6C) and *GATA3* expression (Figure 6D), the Th1 and Th2 master transcription factors, respectively. Using an *in vitro* differentiation system, the induction of classical Th1 and Th2 cells was normal, as defined by expression of Tbet and GATA3 (Figure 6E). However an unusual population was observed in *Nup210*^*KO*^ T cells, where Tbet^+^GATA3^+^ T cells emerged from Th2-polarizing conditions (Figure 6E). At the cytokine production level, a similar phenotype was observed whereby IL-4 production was normal under both Th1- and Th2-polarizing conditions, and IFNγ production was normal in Th1-polarizing conditions, however only Nup210-deficient T cells demonstrated IFNγ production in Th2-polarizing conditions (Figure 6F). Together, these data were suggestive of a pro-inflammatory status of Nup210-deficient mice. An immunological challenge to induce inflammation, using the collagen-induced arthritis model, did not, however, identify any susceptibility to inflammatory disease (Supplementary Figure 7). Together, these results demonstrate that Nup210 restrains IFNγ production under inappropriate, Th2-biased conditions, however the deficiency effect remains subclinical even under inflammatory conditions.

## Discussion

Both the Nup210 mutant mouse strain and the Nup210 knockout mouse strain manifested a disturbance in the peripheral T cells compartment, namely in the ratio of CD4 to CD8 T cells. The observation of general cell biology defects manifesting with T cell components is reoccurring (1), and may lie in the extraordinary rate of proliferating of early stage T cell differentiation in the thymus. Such a model would be consistent with the lethal phenotype observed with Nup210 deficiency in HeLa cells, embryonic *C. elegans* and differentiation embryonic stem cells (23, 26). Indeed, one of the few known functions of Nup210 is the nucleocytoplasmic transport of the mitosis promoting factor (MPF) (27), and it may play a role in the breakdown of the nuclear envelope during mitosis (28, 29). Despite the attractive synergy of such a model, analysis of the thymus suggested normal T cell development, with no alterations in the differentiation stages and no alteration in expression of ThPOK, involved in the CD4 linage commitment or Runx3, involved in the CD8 linage commitment (30, 31). The T cell phenotype, shown here to be T cell intrinsic, instead appears to manifest entirely at the naïve peripheral T cell stage, a relatively quiescent low-activity cell type. This observation, and the observation that cytokine production in activated T cells was altered in Nup210-deficient mice, suggests that the function of Nup210 may lie in gene regulation, such as through altered gene expression (27, 32), rather than in basic cell biology functions such as proliferation. The mechanism by which Nup210 could alter gene expression without entering the nucleus remains unknown (16, 17); the simplest explanation, altered trafficking through the nucleopore, is not supported by experimental testing (32). Here Ptprf was an attractive target, with downregulation observed in post-mitotic myotubes following Nup210 depletion (16), and a known function in T cell biology (33). While this candidate was not observed to be differentially expressed in Nup210-deficient T cells, disturbed gene expression remains an attractive hypothesis for Nup210 function, if only because no obvious alternatives have been proposed. For example, the nuclear envelope protein Lamin A/C alters T cell receptor signaling (34) and plays a role in Th1 differentiation and maintenance by regulation of Tbet and IFNg production (35). The functional role of Nup210 in T cell activation identified here makes the highly specific link between anti-Nup210 autoantibodies and primary biliary cirrhosis (36), a T cell-mediated autoimmune disease (37), even more intriguing.

The most intriguing, and perplexing, finding from the data in this study is the “negative result” of a weak phenotype in Nup210-deficient mice. Indeed, the very finding that Nup210-deficient mice can be generated is highly surprising. Nup210 is a highly conserved component of the nuclear pore, a complex essential for eukaryotic life owing to the function in trafficking between the nucleus and cytoplasm. Previous attempts at generating mice deficient for nucleoporins NUP133, Rae1/Gle2, CAN/Nup214, Nup98, Nup50, Nup96, or Elys were unsuccessful, due to embryonic lethality (19, 38–43). It is feasible, indeed, even likely in light of the current results, that Nup210 is not an obligate component of this complex, and indeed nucleopore-like structures were observed in the knockout mice (Supplementary Figure 3). Compensation by other nuclear pore components is one potential explanation, however it was not observed at the RNA level (Figures 3K,L). Regardless, the deep evolutionary conservation of this protein is difficult to explain without a critical non-redundant function in same aspect of cell biology. Indeed, deficiency in Nup210 in HeLa cells and reduction of Nup210 by RNAi in *C. elegans* resulted in greatly reduced viability and early lethality (23), supporting a critical function for this protein. We document here a function for Nup210 in T cells, however the phenotype induced by deficiency is unlikely to explain the evolutionary conservation, especially across species that do not have T cells. A more likely explanation for the redundancy of Nup210 is that it has a key biological function, driving the deep evolutionary conservation, which is non-essential under laboratory conditions. An example of this are the innate immune sensors, which illustrate both high degrees of conservation and relatively little phenotype in specific-pathogen-free laboratory conditions. Thus Nup210 may have further critical functions which are not revealed in laboratory conditions, but which nevertheless confers a key survival advantage under certain stress conditions. Indeed, shRNA-mediated depletion of Nup210 *in vitro* resulted in the upregulation of endoplasmic reticulum stress-specific caspase cascades (44). Here we tested autoimmune stress (in the context of arthritis induction) and metabolic stress (glucose tolerance after exposure to a high fat diet; data not shown), with no clear phenotype shown, however an infinite range of stress contexts is possible. The generation of these mice opens up the capacity for future exploration of the hidden functions of Nup210.

Afternote: During the review of this paper, an independent study generated *Nup210*^*KO*^ mice, confirming key aspects of our findings (notably, the viability of knockout mice and the T cell-intrinsic defect in peripheral CD4 T cells). Minor differences were observed between the two studies, for example we observed a smaller defect in CD8 T cells, while this was not observed in the Borlido study. In the Borlido study, it was shown that the altered ratio of peripheral CD4 and CD8 T cells was due to the role of Nup210 in the gene regulation of Cav2 and Jun (45). These defects led to the lack of tonic TCR signal transmission and increased expression of FAS, triggering apoptotic cell death of CD4+ T cells in the periphery (45). Our study remains the most comprehensive analysis of thymic development in Nup210-deficient mice and is the first to identify the Th1 bias.

## Materials and methods

### Mice

All animal experiments were approved by the Animal Ethics Committee of the KU Leuven and performed in accordance with the approved protocol. To generate the *Nup210*^*ENUI*476*T*^ strain, founder C57BL/6 male mice were treated with 100 mg/kg ENU and bred to *Foxp3*^*GFP*^ females (46). First-generation (F1) male offspring were bred back to WT females to produce the second-generation (F2) offspring, which were in turn inter-crossed to produce the third generation for phenotypic screening. Phenotypic screening involved flow-cytometric analysis for CD4 and CD8 in the blood. *NUP210*^*KO*^ mice were generated through the European Conditional Mouse Mutagenesis (EUCOMM) program, by insertion of a LacZ/Neo cassette between exons 3 and 5 (Supplementary Figure 1).

### *Ex vivo* flow cytometry

Surface staining was performed in RPMI containing 2% fetal bovine serum and anti-mouse CD16/CD32 Fc block (from hybridoma supernatant generated in-house). The following antibodies were used in this study: CD3 (145-2C11), CD4 (GK1.5 and RM4-5), CD8α (53-6.7), Foxp3 (FJK-16s), IFNγ (XMG1.2), IL-17 (TC11-18H10), IL-4 (BVD6-24G2), IL-2 (JES6-5H4), IL-10 (JES5-16E3), Ly5.1 (A20), Ly5.2 (104) (eBioscience, CA, USA). Nuclear staining for Foxp3 was performed according to the manufacturer's recommendations (eBioScience, CA, USA). Intracellular cytokine staining was performed after a 4-h stimulation with 50 ng/mL phorbol 12-myristate 13-acetate and 0.5 μg/mL ionomycin (Sigma) in the presence of GolgiStop (Monensin A, BD Biosciences, NJ, USA), using Cytofix/Cytoperm (BD Biosciences, NJ, USA). Dead cells were excluded from analysis by staining with Zombie dyes (BioLegend, San Diego, CA, USA) according to the manufacturer's instructions. Samples were analyzed using a BD FACSCanto II instrument (BD Biosciences) and FlowJo software (Treestar Inc., OR, USA).

### Collagen-induced arthritis

CIA was induced in 6–10 week old mice as previously described (47). A total of 100 μL of chick type II collagen (CII, final concentration 1 mg/ml; Sigma, MO, USA) emulsified in complete Freund's adjuvant containing 5 mg/ml heat-killed *M. tuberculosis* H37RA (BD Difco, NJ, USA) was injected intradermally in two sites at the base of the tail. The injections were repeated 21 days later. Animals were monitored three times weekly for erythema and swelling of limbs, and a clinical score (0–3) was given for each paw. Serum was collected using serum separator tubes (Greiner, Vilvoorde, Belgium) and analyzed for anti-chick collagen type II IgG antibodies by ELISA as described (48). Standard curves were constructed from pooled sera of CII hyper-immunized DBA/1 mice, set at 100,000 units/ml.

### Bone marrow chimeras

Red cell-depleted bone marrow from donor mice was depleted for mature T cells by incubation with biotinylated antibodies to CD3, CD4, and CD8 (eBioscience, San Diego, USA) followed by streptavidin-coupled Dynabeads and magnetic separation according to the manufacturer's instructions (ThermoFisher, Gent, Belgium). Bone-marrow chimeras were generated by lethal irradiation (7 Gy for Rag^KO^ recipients, 9.5 Gy for lymphocompetent recipients) of recipients, followed by intravenous (i.v.) injection of 5 × 10^6^ donor cells in saline. Reconstitution was analyzed after 8 weeks by flow cytometry.

### Antigen-specific T cell responses

Wildtype (C57BL/6) and *NUP210*^*KO*^ mice were immunized subcutaneously with 50 ug of 1W1K peptide (EAWGALANKAVDKA, Cambridge Research Biochemicals) emulsified in Incomplete Freund's Adjuvant (F5506, Sigma). Eight days after immunization draining LNs were harvested, processed to single cell suspensions and stained with I-A^b^1W1K tetramer (NIH tetramer bank) for 2 h at room temperature, followed by anti-CD4 (RM4-5, eBioscience) and anti-CD44 (IM7, BioLegend) for 45 min at 4°C. Data were collected on a FACSCantoII (BD Biosciences) and analyzed with FlowJo (Treestar).

### Expression analysis

Quantitative RT-PCR was performed on purified mRNA (Trizol reagent, Ambion, Belgium) from cell populations sorted by flow cytometry from thymi of 6-week old mice, using the GoScript Reverse Transcription kit (Promega, Wisconsin, USA) and FastSYBR green reagents (ThermoFisher, Belgium). The expression of both housekeeping genes hypoxanthine-guanine phosphoribosyltransferase (*Hprt*) and peptidyl-prolyl cis-trans isomerase A (*Ppia*) was used to normalize mRNA expression. PCRs were performed in triplicate. Statistical analysis was performed using Prism (GraphPad). A significance threshold of 5% using non-parametric Mann–Whitney *U*-tests was applied.

Western Blot was performed on thymocytes lysed by sonication in lysis buffer [200 mM NaCl, 50 mM Tris pH 7.5, 1% Triton X-100, 2 mM dithiothreitol, 1 mM EDTA, protease inhibitor (ThermoFisher, Gent, Belgium)]. Lysates (20 ug) were run on 8% NuPAGE BisTris gels and blotted to a polyvinylidene fluoride transfer membrane using the NuPage electrophoresis system (ThermoFisher, Gent, Belgium) according to the manufacturer's recommendations. After washing in NCP (147 mM NaCl, 40 mM Tris pH 8, 0.01% Tween), the membrane was blocked overnight at 4°C with 5% non-fat milk in NCP 0.01% Tween. Primary antibodies against Nup210 (Abcam, Cambridge, UK, ab15600, 1:500) or control vinculin (Sigma-Aldrich, St.Louis, USA, V9131, 1/2000), were incubated in NCP 0.01% Tween, 1% non-fat milk. The membrane was washed in NCP 0.01% Tween and the primary antibody was detected with horseradish peroxidase-conjugated anti-rabbit secondary antibody (ThermoFisher, Gent, Belgium, 1:40,000) for Nup210 and HRP-conjugated anti-mouse secondary antibody (Merck Millipore, Darmstadt, Germany, 1:10,000). After washing in NCP 0.01% Tween, blots were developed using the Amersham ECL Prime Western Blotting Detection Reagent (GE Healthcare, Buckinghamshire, UK). The Spectra multicolor high range protein ladder (ThermoFisher, Gent, Belgium) was used to determine the molecular weights of the visualized bands.

### Nuclear pore imaging

Mouse embryonic fibroblasts were plated onto collagen-coated glass coverslips and cultured overnight to 80% confluency in DMEM high glucose, GlutaMAX, with pyruvate (Thermofisher), supplemented with 15% hyclone fetal calf serum (Thermofisher). Cultures were fixed for 10 min at room temperature with 4% paraformaldehyde in 1 X PBS and washed three times with 1 X PBS. Cells were then blocked for 1 h with 10% normal donkey serum diluted in 1 X PBS also containing 0.25% TritonX-100, followed by overnight incubation at 4°C with Monoclonal Antibody 414 (Mab414; AbCam ab24609) diluted 1:50 in the same solution. The following day coverslips were briefly rinsed in 1 X PBS, washed 3 x 10 min with 1 X PBS, and then incubated for 1 h at room temperature with Alexa Fluor 488 anti-mouse (Jackson Immunoresearch) in the 10% normal donkey serum solution. A second set of washes were applied and then coverslips were inverted and mounted onto slides with Vectashield containing DAPI (Vector Laboratories). For imaging a Nikon (Tokyo, Japan) C2 confocal scanhead attached to a Nikon TiE inverted microscope outfitted with a APO 60x 1.4 NA oil objective lens was used. Images of different genotypes were processed identically using ImageJ and Photoshop.

### *In vitro* assays

The conditions for *in vitro* suppression of CD4 T cell proliferation by Tregs were adapted from Collison and Vignali (49). Briefly, CD4^+^CD25^+^ Treg were flow sorted from WT and *Nup210*^−/−^ mice on a BD Aria I. Conventional CD4^+^Foxp3^−^ T cells (Tconv) were sorted from CD45.1 Foxp3-Thy1.1 reporter mice (50). Tconv (10^5^) were labeled with CellTrace Violet (Invitrogen), and were co-cultured with varying ratios of unlabeled Treg. Proliferation was stimulated by the addition of Rag1^−/−^ splenocytes (5 × 10^4^) and anti-CD3 (0.25 μg/ml, clone 145-2C11, eBioscience). After 5 days, the cells were stained for CD4-PE-Dazzle594 (clone GK1.5, BioLegend), Thy1.1-APC (clone HIS51, eBioscience), CD45.1-PE-Cy7 (clone A20, eBioscience), and fixable viability dye eFluor780, and the proliferation of the viable CD45.1^+^CD4^+^ Tconv was assessed by measuring the dilution of the CellTrace Violet. Proliferation was calculated by comparing the median intensity of the CellTrace Violet in the test conditions with the median for undivided cells.

For Th1/Th2 induction, naïve T cells were purified by negative selection using the Naïve CD4+ T cell Isolation Kit (Miltenyi Biotec) according to manufacturer's recommendation. Cells were cultured with 10 μg/ml anti-CD3 and 2.5 μg/ml anti-CD28 (eBioscience) for 6 days, under either Th1 (10 ng/ml 1L-12 and 10 μg/ml anti-IL-4; eBioscience) or Th2 conditions (10 ng/mL IL-4, Biolegend, and 10 μg/mL anti-IFN-γ, eBioscience). Cells were probed for transcription factor expression at 3 days and cytokine expression at 6 days. Transcription factor staining was performed after fixation and permeabilization (Foxp3/Transcription Factor Staining Buffer, eBioscience), with anti-GATA3 (eBioscience, AB_1963600), and anti-T-bet (eBioscience, AB_2686976). Cytokine expression staining was performed after culture with Phorbol-12,13-dibutyrate (1 μg/mL, Tocris Bioscience), Ionomycin calcium salt (1.5 μg/mL Tocris Bioscience) and Brefeldin A (4 μg/mL, Tocris Bioscience), and fixation with Foxp3/Transcription Factor Staining Buffer (Thermofisher). Cells were stained with anti-IL-4 (eBioscience, AB_469494) and anti-IFNγ (eBioscience, AB_469680). Samples were analyzed using a BD FACSCanto II instrument (BD Biosciences) and FlowJo software (Treestar Inc., OR, USA).

## Author contributions

AN, OB, PL, AD, AC, BM-D, and BC performed the experiments. AN analyzed the data. REG, MAL, and AL supervised experiments. AL and SH-B designed and led the study. AL and AN wrote the manuscript.

### Conflict of interest statement

The authors declare that the research was conducted in the absence of any commercial or financial relationships that could be construed as a potential conflict of interest.

## Supplementary material

Supplementary Figure 1 Splenic T cell populations in ENU mutant mice and F2 mice. Spleens from 6 to 8 week old mice were analyzed by flow cytometry for CD4^+^ and CD8^+^ T cell subsets. **(A)** ENU mice: Absolute numbers of CD4^+^Foxp3^+^ Treg/spleen (*n* = 6, 10). **(B)** F2 progeny gest: %CD4 and CD8 of splenic lymphocytes (*n* = 14, 7, 9). Mean ± SEM, with individual data points (*t-*test).

Supplementary Figure 2 Generation of Nup210-deficient mice. Schematic depiction of *Nup210* locus design in original EUCOMM ES cells.

Supplementary Figure 3 Nuclear pore complexes are present in NUP210 knockout cells. Images of mouse embryonic fibroblasts labeled with nuclear pore complex antibody “MAb414” (green) and DAPI (blue). **(A,B)**. Confocal images that bisect the nucleus show MAb414 labeling at the nuclear periphery of WT and NUP210 knockout cells. **(C,D)** Confocal images that scan the nuclear surface show abundant punctate MAb414 labeling. The density and distribution of MAb414 punctate foci appear similar between WT and NUP210 KO nuclei (far right panels). Images are representative of observations from three wildtype and three NUP210 KO fibroblast cell lines.

Supplementary Figure 4 *Nup210*^*KO*^ mice have normal splenic myeloid populations. **(A)** Representative flow cytometry plots for the gating strategy for myeloid cells. **(B)** Summarized data for the splenic myeloid compartment of WT and *Nup210*^*KO*^ mice (*n* = 3, 11, 8, Mean ± SEM).

Supplementary Figure 5 *Nup210*^*KO*^ mice have normal splenic B cell populations. **(A)** Representative flow cytometry plots for the gating strategy for B cell populations. **(B)** Summarized data for the splenic B cell compartment of WT and *Nup210*^*KO*^ mice (*n* = 7, 9, 8. Mean±SEM.).

Supplementary Figure 6 Representative flow cytometry of WT and NUP201KO spleens. Spleens from 6 to 11 week old wildtype and *Nup210*^*KO*^ mice were analyzed for T cell subsets using flow cytometry. Representative plots shown.

Supplementary Figure 7 Normal susceptibility to collagen-induced arthritis in Nup210 knockout mice. *Nup210*^*het*^ and *Nup210*^*KO*^ mice were immunized with chick type II collagen (CII) on day 0 and boosted on day 21. Disease incidence and severity was monitored 3 times weekly up to day 45 (*n* = 8, 6). **(A)** Total disease score per mouse (maximum 3 per paw). No significant difference by *T*-test comparing the area under the curve calculated for individual mice. **(B)** Incidence of disease (not significantly different, Log-rank Mantel-Cox test). **(C)** Anti-CII IgG2a levels in the serum on day 45 (not significantly different). **(D)** CD4:CD8 T cell ratio in the spleen on day 45, as measured by flow cytometry. Mean ± SEM, with individual data points.
