# Supplementary figures and images for: Mice Deficient in Nucleoporin Nup210 Develop Peripheral T Cell Alterations
Source: Front Immunol. 2018 Sep 28;9:2234. doi: 10.3389/fimmu.2018.02234 (PMC6173157; doi:10.3389/fimmu.2018.02234)

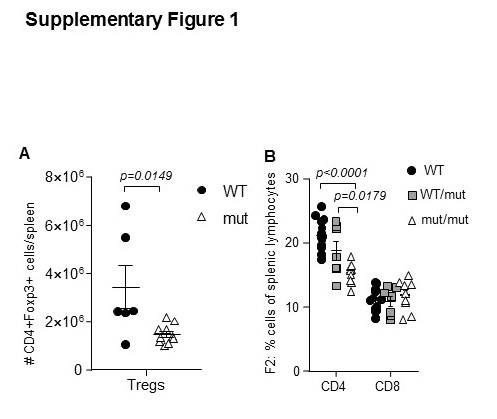

Supplement: Supplementary Figure 1 — Splenic T cell populations in ENU mutant mice and F2 mice. Spleens from 6 to 8 week old mice were analyzed by flow cytometry for CD4+ and CD8+ T cell subsets. (A) ENU mice: Absolute numbers of CD4+Foxp3+ Treg/spleen (n = 6, 10). (B) F2 progeny gest: %CD4 and CD8 of splenic lymphocytes (n = 14, 7, 9). Mean ± SEM, with individual data points (t-test). [file Image_1.JPEG]

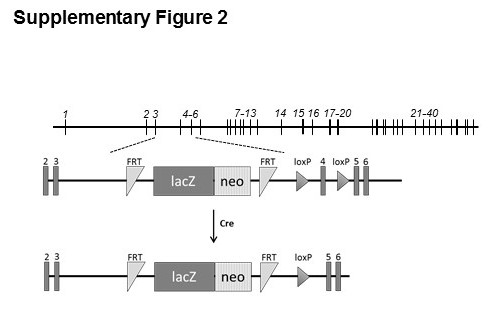

Supplement: Supplementary Figure 2 — Generation of Nup210-deficient mice. Schematic depiction of Nup210 locus design in original EUCOMM ES cells. [file Image_2.JPEG]

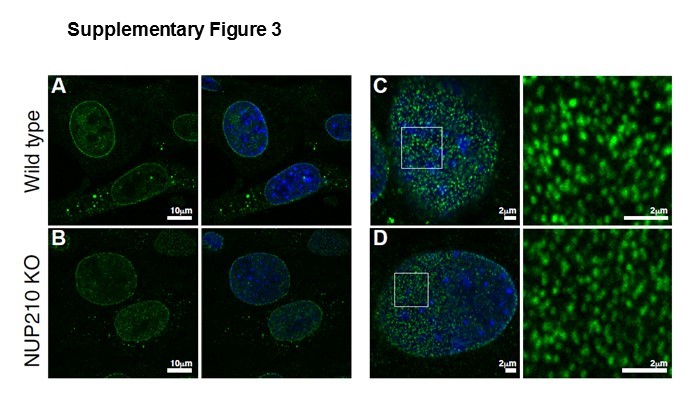

Supplement: Supplementary Figure 3 — Nuclear pore complexes are present in NUP210 knockout cells. Images of mouse embryonic fibroblasts labeled with nuclear pore complex antibody “MAb414” (green) and DAPI (blue). (A,B). Confocal images that bisect the nucleus show MAb414 labeling at the nuclear periphery of WT and NUP210 knockout cells. (C,D) Confocal images that scan the nuclear surface show abundant punctate MAb414 labeling. The density and distribution of MAb414 punctate foci appear similar between WT and NUP210 KO nuclei (far right panels). Images are representative of observations from three wildtype and three NUP210 KO fibroblast cell lines. [file Image_3.JPEG]

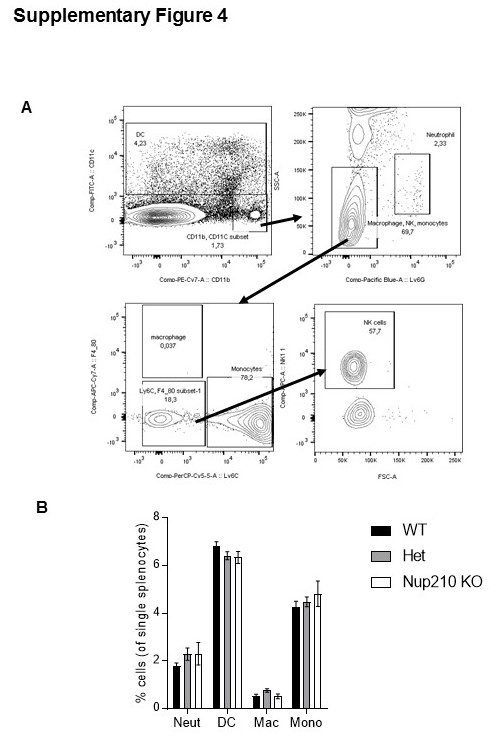

Supplement: Supplementary Figure 4 — Nup210KO mice have normal splenic myeloid populations. (A) Representative flow cytometry plots for the gating strategy for myeloid cells. (B) Summarized data for the splenic myeloid compartment of WT and Nup210KO mice (n = 3, 11, 8, Mean ± SEM). [file Image_4.JPEG]

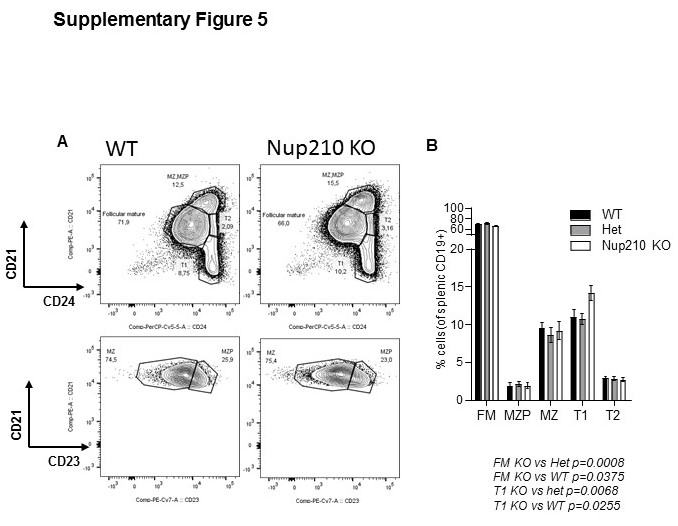

Supplement: Supplementary Figure 5 — Nup210KO mice have normal splenic B cell populations. (A) Representative flow cytometry plots for the gating strategy for B cell populations. (B) Summarized data for the splenic B cell compartment of WT and Nup210KO mice (n = 7, 9, 8. Mean±SEM.). [file Image_5.JPEG]

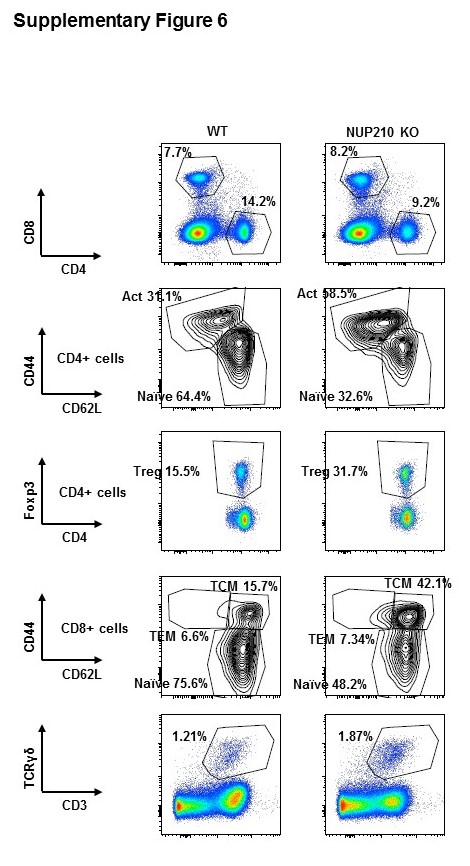

Supplement: Supplementary Figure 6 — Representative flow cytometry of WT and NUP201KO spleens. Spleens from 6 to 11 week old wildtype and Nup210KO mice were analyzed for T cell subsets using flow cytometry. Representative plots shown. [file Image_6.JPEG]

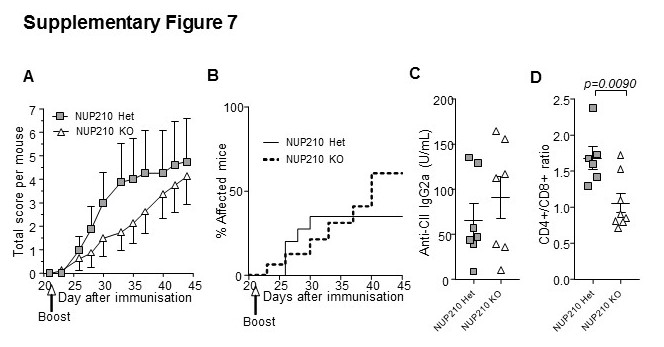

Supplement: Supplementary Figure 7 — Normal susceptibility to collagen-induced arthritis in Nup210 knockout mice. Nup210het and Nup210KO mice were immunized with chick type II collagen (CII) on day 0 and boosted on day 21. Disease incidence and severity was monitored 3 times weekly up to day 45 (n = 8, 6). (A) Total disease score per mouse (maximum 3 per paw). No significant difference by T-test comparing the area under the curve calculated for individual mice. (B) Incidence of disease (not significantly different, Log-rank Mantel-Cox test). (C) Anti-CII IgG2a levels in the serum on day 45 (not significantly different). (D) CD4:CD8 T cell ratio in the spleen on day 45, as measured by flow cytometry. Mean ± SEM, with individual data points. [file Image_7.JPEG]
